# Predictive Value of an Early Diagnosis of Orthostatic Hypotension on Cardiovascular Events

**DOI:** 10.1111/jch.70018

**Published:** 2025-02-17

**Authors:** Fosca Quarti‐Trevano, Cesare Cuspidi, Guido Grassi

**Affiliations:** ^1^ Department of Medicine and Surgery University of Milano‐Bicocca Milano Italy

**Keywords:** cardiovascular risk, orthostatic hypotension, sympathetic nervous system

1

A large number of prospective studies carried out during the past 20 years, some of them included in a recent meta‐analysis, have conclusively shown that clinically overt orthostatic hypotension represents a pathological condition associated with an increase in fatal and non‐fatal cardiovascular events and in all‐cause mortality [1, 2, 3, 4, 5, 6, 7, 8]. Whether and to what extent this is the case in the advanced full manifest clinical condition only or it is also detectable in the initial clinical phases of the disease is much less clearly defined, however.

In the present issue of the *Journal*, a group of Chinese investigators reports the results of a retrospective study [9] aimed at investigating the occurrence of major adverse cardiovascular events (MACE), defined as cardiovascular death, myocardial infarction, angina pectoris, heart failure, or atrial fibrillation in patients aged more than 50 years with a diagnosis of an initial orthostatic hypotensive condition and followed, on average, for more than 5 years. Early diagnosis was founded on the detection of a transient decrease in systolic blood pressure of magnitude greater than 40 mmHg and/or in diastolic blood pressure greater than 20 mmHg within 15 s of active standing, with a blood pressure recovery between 15 s and 3 min of standing [10]. A group of patients with a full‐blown orthostatic hypotensive disease served as a control to compare the data collected in the patients displaying the initial stage of the clinical condition. Results show that not only sustained but also initial orthostatic hypotension increases the risk of cardiovascular events, the mortality risk is, however, augmented in the advanced clinical condition only.

Several intriguing findings of the study deserve to be mentioned and discussed. First, the study makes the diagnosis of orthostatic hypotension on the basis of the blood pressure responses to standing detected during the initial 15 s of the maneuver. Does this approach guarantee an accurate and careful assessment of the disease? Based on current guidelines [10], the approach seems accurate enough, given the notion that the circulatory responses characterizing the first 15 s of the maneuver are more likely to detect the blood pressure drop in this very initial time period. To assess such short‐lasting blood pressure changes appropriately, the Authors correctly have made use of the continuous non‐invasive arterial blood pressure monitoring device, which allows to properly detect short‐lasting blood pressure changes occurring in this temporal window of very short duration. Another issue, which is important from a methodological but also a diagnostic viewpoint, refers to the within‐subject variability of the hemodynamic responses to standing [11, 12]. In other words, should the diagnosis of the condition be based only on a single evaluation or should it be based on the average of the responses repeated 2–3 times in a reasonable time window? The question has no answer, given the fact that the diagnosis in the studies performed so far is founded only on the evaluation of the hemodynamic response to a single maneuver.

Secondly, the Authors reported that antihypertensive drug treatment was one of the factors included in the multivariable analysis of the data involved in the occurrence of an initial or sustained form of orthostatic hypotension, in conjunction with other hemodynamic and non‐hemodynamic factors. Specifically, the number of antihypertensive medications was greater in the sustained orthostatic hypertensive group as compared to the patients displaying the initial clinical form. Also, the presence of the various antihypertensive drug classes was significantly different between groups. This was particularly the case for the angiotensin‐converting enzyme (ACE) inhibitor drugs, which were used in a significantly less fraction of the patients affected by the initial form of the disease than in controls. Taking into account that this class of drugs has been shown to be protective from orthostatic hypotension [13], this finding may represent an important therapeutic difference.

A further study result that deserves to be briefly discussed refers to the finding that neither in the group of patients with overt orthostatic hypotension nor in the one including patients in the initial clinical phases of the disease resting heart rate values were elevated. This finding substantially differs from the majority of data reported in various studies showing elevated resting heart rate values in patients with orthostatic hypotension [1, 2, 3, 4, 5, 6, 7, 8] and may be related to a greater use of drugs reducing heart rate values, such as beta‐blockers, in the study populations. Indeed the evidence of elevated heart rate values assessed in the resting state underlines the central role exerted by the autonomic nervous system for the cardiovascular adjustments to postural changes and emphasizes the relevance of the autonomic neurogenic factors in the pathophysiology of the disease [11, 12]. From a mechanistic view point, an elevated heart rate reflects an increased sympathetic cardiac drive at the level of the sinus node coupled with a reduced inhibitory vagal input to the same cardiac region [14]. An elevated heart rate value also means, from an operative point of view, a lesser ability of heart rate to increase further in response to a given maneuver or intervention. Since during orthostatic stress the heart rate increase is of vital importance for maintaining stable blood pressure (particular systolic) [14], its attenuated increased during the maneuver may make the patient more prone to develop postural hypotension. In contrast, since the behavior of diastolic blood pressure during orthostatic stress is less dependent on heart rate [14], an impairment of the ability of sympathetic neural function to trigger vasoconstriction may be suggested to occur in these patients. The results of the present study by showing superimposable heart rate values in healthy control subjects and in patients with an initial or a sustained clinical form of the disease may also indicate the limitations of this hemodynamic variable in reflecting autonomic (particularly sympathetic) cardiovascular drive [15]. Indeed, in a number of different diseases, such as essential hypertension, chronic heart failure, renal insufficiency, obesity, and diabetes mellitus we found that not always heart rate values reflect the sympathetic overdrive characterizing the above mentioned clinical conditions and directly documented by the microneurographic recording of muscle sympathetic nerve traffic [16, 17, 18, 19, 20].

Finally, it should be worthy of mention that another clinical phenotype of increasing clinical relevance, involving such as orthostatic hypotension the blood pressure alterations characterizing the assumption of the upright posture, is represented by the opposite condition, namely orthostatic hypertension [21]. This clinical hypertensive phenotype, defined as an exaggerated orthostatic pressor response associated with systolic blood pressure of at least 140 mmHg while standing, has also been shown to be associated with an increase in all‐cause and cardiovascular mortality [21]. Future studies will be needed to determine whether, similar to what has been reported in the orthostatic hypotension study commented on in the present editorial [9], also the orthostatic hypertensive phenotype may display an increased cardiovascular risk both in its advanced and initial clinical phenotypes.
